# STAT3-Mediated Metabolic Reprograming in Cellular Transformation and Implications for Drug Resistance

**DOI:** 10.3389/fonc.2015.00121

**Published:** 2015-06-08

**Authors:** Valeria Poli, Annalisa Camporeale

**Affiliations:** ^1^Department of Molecular Biotechnology and Health Sciences, Molecular Biotechnology Center, University of Torino, Torino, Italy

**Keywords:** STAT3 transcription factor, metabolism, drug resistance, malignant transformation, mitochondria

## Abstract

Signal transducer and activator of transcription (STAT)3 mediates the signaling downstream of cytokine and growth factor receptors, regulating the expression of target genes. It is constitutively phosphorylated on tyrosine (Y-P) in many tumors, where its transcriptional activity can induce a metabolic switch toward aerobic glycolysis and down-regulate mitochondrial activity, a prominent metabolic feature of most cancer cells, correlating with reduced production of ROS, delayed senescence, and protection from apoptosis. STAT3 can, however, also localize to mitochondria, where its serine-phosphorylated (S-P) form preserves mitochondrial oxidative phosphorylation and controls the opening of the mitochondrial permeability transition pore, also promoting survival and resistance to apoptosis in response to specific signals/oncogenes such as RAS. Thus, downstream of different signals, both nuclear, Y-P STAT3, and mitochondrial, S-P STAT3, can act by promoting cell survival and reducing ROS production. Here, we discuss these properties in the light of potential connections between STAT3-driven alterations of mitochondrial metabolism and the development of drug resistance in cancer patients.

## Introduction

Signal transducer and activator of transcription (STAT)3 becomes activated in response to cytokines, growth factors, and oncogenes, via phosphorylation on its tyrosine 705 residue (Y-P) mediated by receptor-associated JAK kinases. Y-P STAT3 concentrates into the nucleus, where it binds to gene promoters modulating their transcription (1). Being expressed almost ubiquitously and activated by a wide variety of signals, it is perhaps not surprising that STAT3 can activate cell-specific repertoires of target genes, thus exerting cell- and context-specific functions (2). For example, STAT3 can trigger induction of acute phase genes during inflammation, liver regeneration, proliferation of B lymphocytes, terminal differentiation and growth arrest in monocytes, lysosome-mediated apoptosis in the involuting mammary gland, as well as maintenance of embryonal stem cells pluripotency (2).

These pleiotropic functions may also have to do with the differential activities of its post-translationally modified forms. First, STAT3 can also be phosphorylated on serine 727 (S-P), with both stimulating and inhibitory effects on transcription (3–6), and with a prominent role in regulating mitochondrial activities [see below (7, 8)]. Second, STAT3 acetylation by the p300 co-activator can enhance dimer stability and transcriptional activity and promote its interaction with DNA methyl transferase 1, leading to hypermethylation of target oncosuppressor promoters (9). Finally, STAT3 activity can also be positively or negatively regulated by methylation, depending on the residue involved (10, 11). STAT3 activation is tightly controlled by a number of negative regulators, including phosphatases, suppressor of cytokine signaling proteins (mainly SOCS3), and protein inhibitor of activated STAT (PIAS) proteins, in particular, PIAS3 (12, 13).

Signal transducer and activator of transcription 3 is considered as an oncogene, being persistently activated by Y-P in more than 50% of human tumors of both solid and hematological origin, which often become addicted to its activity (14). Aberrant phosphorylation is mainly due to persistent activity of its upstream activators or to disruption of the negative control mechanisms (14), although activated mutations have also been described, mainly found in exons 20 and 21, encoding for the SH2 domain (15–20). Interestingly, also acetylation and S-P can be constitutively activated in tumors (4, 9, 21).

A direct role of STAT3 in oncogenesis was first demonstrated by overexpressing a constitutively active STAT3 mutant form, STAT3C, which shows greatly increased function in cells under basal conditions and is hypersensitive to IL-6 stimulus (22). Its overexpression leads to malignant transformation in immortalized fibroblasts and epithelial cells (23). Further to this finding, we have shown that primary mouse embryonal fibroblasts (MEFs) expressing physiological levels of STAT3C undergo spontaneous transformation when immortalized via the 3T3 protocol, suggesting that a weak but continuous STAT3 activity can act as a first hit in tumor transformation (24). This is particularly relevant in the context of inflammation-induced cancer, where STAT3 is known to play a major role, consistent with the observation that persistent IL-6 production and STAT3 activation are prominent features of chronic inflammation (25). Indeed, STAT3-dependent tumor transformation usually correlates with enhanced expression of anti-apoptotic and pro-proliferative genes such as Bcl-2, MCL-1, cyclin-D1, and c-myc, which help preventing apoptosis and stimulating tumor growth, migration, and invasion (14). Importantly, many activated oncogenes including vSRC and RAS require STAT3 to elicit tumor transformation (8, 26).

In addition to these “canonical” functions, it has become increasingly evident that STAT3 is also a regulator of cell energy metabolism, which can heavily impact on tumor transformation and growth. Both nuclear and mitochondrial STAT3 are involved in these metabolic activities, as outlined below.

## Nuclear STAT3, Energy Metabolism, and Cell Transformation

**Figure 1:**
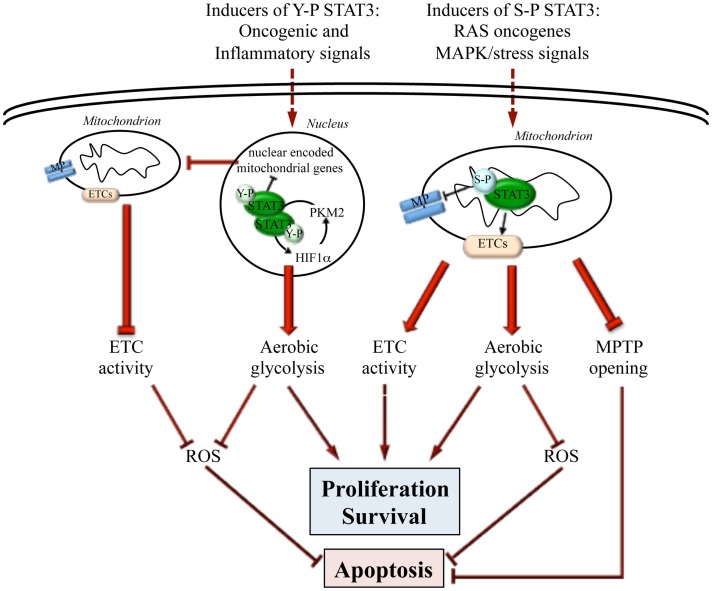
**Differential actions of nuclear and mitochondrial STAT3**. STAT3 can influence energy metabolism both from within the nucleus and the mitochondrion, depending on specific post-transcriptional modifications (Y-P or S-P) triggered by different oncogenic stimuli. Y-P nuclear STAT3 mediates transcriptional up-regulation of HIF-1α and the down-regulation of mitochondrial genes. This leads to enhanced aerobic glycolysis, blunted ETC activity, and decreased ROS production, thus promoting fast proliferation while inhibiting apoptosis. On the other hand, also S-P STAT3 mitochondrial activity leads to enhanced cell proliferation and survival and to apoptosis resistance by preserving ETC activity, stimulating aerobic glycolysis, decreasing ROS production, and inhibiting the opening of the mitochondrial permeability transition pore (MPTP).

We have recently reported that low but constitutive STAT3 transcriptional activity in MEFs expressing the STAT3C form can trigger a metabolic switch by enhancing aerobic glycolysis and reducing oxidative phosphorylation and mitochondrial activation (27) (Figure 1), thus mimicking a common metabolic feature of tumor cells known as the Warburg effect (28). This activity contributes to STAT3 pro-oncogenic functions, since it is required for survival and *in vivo* growth of STAT3-addicted human cancer cell lines, which also display low but constitutive Y-P STAT3. The effects on glycolysis are mainly mediated by chronically increased HIF-1α expression. Indeed, there are several important connections between STAT3 and the hypoxia sensor HIF-1α. First, STAT3 constitutive activity was shown to directly up-regulate *Hif-1*α transcription in melanoma cells (29), and to increase HIF-1α protein levels in several tumor cell types (e.g., breast, kidney, ovary, prostate, melanoma), correlating with EMT and invasion (30–33). Second, STAT3 can cooperate with HIF-1 by binding to its responsive promoters, ensuring the formation of a transcriptionally active complex (34, 35). Finally, STAT3 appears to be involved in a feed forward loop that leads to enhanced aerobic glycolysis and fast proliferation: oxygen deprivation or oncogenes, up-regulating HIF-1α and increasing HIF-1 activity, lead to increased levels of the pyruvate kinase PKM2 isoform; in turn, this enhances HIF-1 transcriptional activity and directly phosphorylates STAT3 (36, 37); closing the loop, activated STAT3 up-regulates HIF-1α expression (38) (Figure 1).

Despite a well-accepted pro-tumorigenic role, STAT3 can also exert tumor-suppressor activities (39–41), and was reported to negatively regulate HIF-1α protein levels and aerobic glycolysis under hypoxic conditions in a model of thyroid cancer (39), suggesting tissue and context specificity of the mechanisms described above.

Metabolic activities of STAT3 and HIF-1α may be also co-regulated by sirtuins, a family of highly conserved NAD^+^-dependent deacetylases that act as cellular sensors to detect energy availability and modulate metabolic processes. In particular, SIRT1 regulates STAT3 acetylation (42), while SIRT3 destabilizes HIF-1α and its target genes (43). STAT3 was shown to enhance glucose release by hepatocytes by inhibiting the transcription of *PEPCK1* and *g6pase* (44, 45), thus suppressing gluconeogenesis, and SIRT1-dependent STAT3 deacetylation disrupts this inhibitory effect (46). The down-regulation of SIRT-1 expression often observed in cancer (47) may thus contribute to maintain STAT3 activity. Indeed, human and murine hepatocellular carcinomas that show down-regulation of SIRT1 display significantly reduced expression of gluconeogenic enzymes and increased release of glucose into circulation, due to the activation of an IL-6-STAT3 signaling pathway leading to the up-regulation of miR-23a (48). This in turn down-regulates the expression of gluconeogenic enzymes such as PGC1α and G6PC. The consequent accumulation of glucose intermediates is likely used by tumors to sustain rapid proliferation. Moreover, since PGC1α positively regulates mitochondrial biogenesis and respiration, its STAT3-dependent down-regulation may also contribute to decrease mitochondrial activity (49, 50).

Increased glycolysis and decreased mitochondrial activity might help reducing ROS production, as it is indeed observed in STAT3C MEF cells (27), thus delaying cell senescence and enhancing cell survival. Additionally, in neuronal cells, STAT3 was shown to regulate SOD2 expression, increasing the scavenge of superoxide radicals (51). Interestingly, low-ROS levels are known to correlate with fast tumor cell proliferation (52).

Not least, the effects of STAT3 on glucose metabolism may also be partly mediated by c-myc, a well-known direct transcriptional target that up-regulates glycolysis genes such as GLUT-1, HK2, ENO-1, and PFKM (53, 54).

## Mitochondrial STAT3, Energy Metabolism, and Cell Transformation

Signal transducer and activator of transcription 3 can localize to mitochondria, mainly in the matrix, where its S-P form can regulate mitochondrial functions independently from its transcriptional activity (7, 8, 55). Mitochondrial transport was proposed to involve the interaction with GRIM-19 (56), a cell death regulatory protein that is an essential component of respiratory chain complex I (57). Interestingly, STAT3 was shown to mediate cell death upon TNF-induced necroptosis, which triggers S-P STAT3 through RIPK1 activity, its interaction with GRIM-19 and the accumulation of the complex in mitochondria, where it leads to increased ROS production and cell death (58). Other import mechanisms have been proposed, involving the activities of the heat shock protein H11 kinase/Hsp22, a potential component of organelle import (59), and of the import receptor subunit Tom20 (55).

Mitochondrial STAT3 is involved in maintaining optimal oxidative phosphorylation levels in cardiac and nerve cells as well as in RAS-transformed tumor cells (7, 8, 60–62). Indeed, STAT3 deletion results in a significant reduction of complex I and II activities in murine hearts, which can be rescued by expressing a mitochondrially targeted STAT3 (7). Accordingly, upon induction of cardiac ischemia, STAT3 protects complex I-dependent respiration from injury, decreasing cytochrome *c* release and ROS production (63). Mitochondrial STAT3 was proposed to act by interacting with respiratory complexes I, II, and V (7, 8), and is able to improve complex I respiration and calcium retention even in isolated mitochondria of post-conditioned hearts (64). Additionally, STAT3 inhibits the opening of the mitochondrial permeability transition pore (MPTP) by interacting with cyclophilin D, thus inhibiting apoptosis through blockade of MPTP-mediated cytochrome *c* release (55) (Figure 1).

The connections between mitochondrial STAT3 activities and ROS production are somewhat contradictory. NGF-dependent STAT3 S-P results in increased mitochondrial STAT3 localization and higher ROS production in neuronal cells, leading to faster neurite outgrowth (61). By contrast, STAT3-deficient astrocytes produce high levels of ROS, decrease glutathione concentration and are unable to maintain mitochondrial membrane potential and cell viability (60), although whether these effects require cytoplasmic or nuclear STAT3 activity has not been determined.

A number of reports suggest that S-P STAT3 can contribute to tumor transformation and growth in several malignancies, including chronic lymphocytic leukemia, myeloproliferative neoplasms (65), prostate, and breast cancer (21, 66, 67). Indeed, mitochondrial S-P STAT3 is required for tumor transformation mediated by oncogenic RAS, favoring both aerobic glycolysis and ETC activity and increasing ATP abundance (8). Although RAS oncogenes can activate several serine kinases able to phosphorylate STAT3 on S727, the MEK-ERK pathway appears to play a prominent role since it was shown to be necessary for S-P of mitochondrial STAT3 and RAS-mediated transformation (62) (Figure 1).

Mitochondrial S-P STAT3 enhances growth and invasion of the murine 4T1 breast cancer cells, both *in vitro* and *in vivo*, by increasing complex I coupling and reducing ROS production and apoptosis (68). In addition, a single nucleotide germline polymorphism (SNP) in the FGFR4, which has been linked to enhanced pituitary tumorigenesis, is associated with increased S-P STAT3, supporting a pro-tumorigenic role of aberrantly regulated mitochondrial STAT3 (69).

A link between mitochondrial STAT3 and tumorigenesis has also been suggested in skin cancer. Treatment of keratinocytes with the TPA tumor promoter results in increased mitochondrial S-P STAT3 via a PKCε-dependent mechanism (70). Interestingly, under these conditions STAT3 was shown to bind to and regulate mitochondrial DNA transcription, suggesting that perhaps STAT3 accumulation in mitochondria may impact on the transcription of mitochondrial-encoded genes also in other circumstances.

Despite several reports suggesting that STAT3 may act by interacting with ETC components, this mechanism is still controversial, mainly due to the disproportioned stoichiometry between STAT3 and ETC proteins (71). It was also noted that ectopically expressed, fluorescent-tagged recombinant STAT3 fusion protein cannot be visualized in association with mitochondria by live cell imaging (72). Taken together, these findings suggest that mitochondrial STAT3 may perhaps act catalytically rather than structurally.

In conclusion, similar to its transcriptionally active counterpart, mitochondrial STAT3 is potentially able to regulate cellular metabolism to warrant cell survival to apoptotic stimuli upon different kinds of stress such as, for example, cardiac ischemia or oncogenic transformation. STAT3 appears therefore to act as a hub integrating multiple signals, which lead to its Y-P or S-P, or both, at the level of energy metabolism and apoptosis control. As disruption of energy metabolism is a common feature of all tumor cells, this central metabolic role of both Y-P and S-P STAT3 may well explain the addiction to STAT3 shown by so many biologically distinct tumors.

## STAT3 and Drug Resistance

Most common chemo- or radio-therapeutic agents trigger cell damage and the activation of the intrinsic apoptotic pathway, leading to cell death (73, 74). In particular, oxidative damage triggered by enhanced ROS accumulation is a prominent effect of both ionizing radiation and pharmacological agents such as gemcitabine, cisplatin, doxorubicin, and elesclomol (75). Cell death is brought about by the activation of p53 and of pro-apoptotic members of the Bcl-2 family, leading to changes in the inner mitochondrial membrane that result in the loss of transmembrane potential and release from the mitochondrial intermembrane space of soluble and cytotoxic proteins such as cytochrome *c*, the Smac/DIABLO complex, nucleases, and proteases. In turn, pro-apoptotic proteins activate caspases that mediate cell destruction via several pathways (76, 77).

Both in normal and in chemotherapy-sensitive tumor cells, stimulation of pro-apoptotic factors tilts the life/death balance toward death. However, the balance between pro- and anti-apoptotic proteins is often compromised in tumor cells, due to the aberrant regulation of apoptosis-modulating pathways and enhancing survival. Indeed, a great percentage of cancer patients fail to respond to chemo- and/or radio-therapy, or experience tumor relapse due to the expansion of drug-resistant tumor cell clones, thus limiting the long-term efficacy of current therapies (78).

Distinct mechanisms can contribute to the development of drug resistance, including (i) alterations of drug metabolism, which determine increased efflux, decreased uptake, enhanced detoxification, and sequestration. Particularly prominent is the enhanced efflux determined by increased activity of the P-glycoprotein encoded by the multidrug resistance (MDR)-1 gene and of other MRP pumps; (ii) decreased drug activation; (iii) modification of drug targets with activation of compensatory signaling receptors or effectors, by either gene mutation or amplification; (iv) feedback loops that are activated following drug-mediated inhibition of pro-tumorigenic targets, associated with up-regulation of alternative RTKs that in turn sustain tumor proliferation; and, finally, (v) dysregulation of apoptotic pathways (79–82).

**Figure 2:**
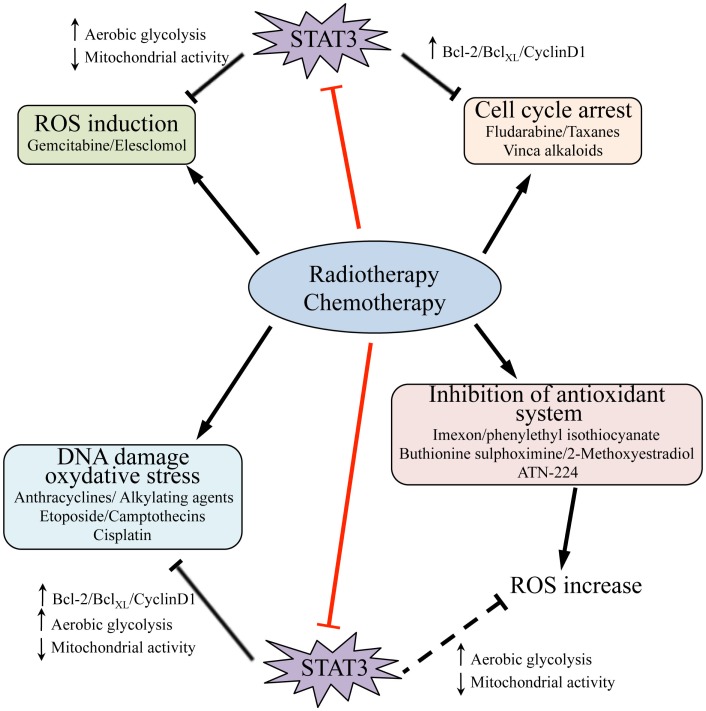
**Signal transducer and activator of transcription 3 activities and resistance to radio- and chemotherapy**. Chemotherapy as well as radiation therapy trigger cell death (*black arrows*) by inducing cell cycle arrest and promoting DNA damage and, as consequence, oxidative stress; they can also induce ROS production by different means, including inhibition of the endogenous antioxidant systems. STAT3 activity contributes to resistance of tumor cells to these treatments, and accordingly some chemical agents act at least partly by directly targeting STAT3 (i.e., anthracyclines, butyrate, sulindac, curcumin, cucurbitacin, *red bars*). STAT3 activity may counteract the action of radiation and pharmacological compounds by mediating transcription of pro-survival and cell cycle genes, such as Bcl-2, Bcl_XL_, and cyclin-D1, and by promoting increased aerobic glycolysis while decreasing mitochondrial activity and ROS production (*black bars*). It is likely that STAT3-mediated down-regulation of ROS may also overcome the action of several agents that impact on the activity of endogenous antioxidant systems (*dashed black bar*). Thus, therapeutic strategies involving the use of inhibitory molecules directed against STAT3, and particularly targeting its mitochondrial functions, hold promise for reverting cancer cells drug resistance.

Multiple STAT3 activities have been correlated with drug resistance in cancer (Figure 2). Indeed, the levels of transcriptionally active Y-P STAT3 are often elevated in drug-resistant cancer cells (83, 84). STAT3 may enhance resistance to conventional chemo- and radio-therapies by inducing the expression of survival proteins and cell cycle genes, which are well-known STAT3 targets (as Bcl-2, survivin, c-myc, cyclin-D1, and Mcl-1), and by down-regulating tumor-suppressor genes either directly, like p53, or indirectly via ZEB1 induction (85–92). Indeed, several reports correlate high levels of tumor-secreted cytokines, such as IL-6 and IL-10, with STAT3-mediated activation of anti-apoptotic factors and drug resistance (93–98). These observations have prompted efforts to exploit the beneficial effects of STAT3 inhibitors to synergistically enhance the efficacy of chemotherapeutic agents. Indeed, several commonly used compounds including anthracyclines, butyrate, sulindac, curcumin, and cucurbitacin have been proposed to owe their anti-tumoral effects at least partly to their ability to directly down-modulate STAT3 activity (99). Moreover, specific STAT3 inhibitors acting at different levels have been tested alone or in combination with chemical agents. For example, treatment of nasopharyngeal carcinoma cell lines, widely insensitive to cisplatin and to radiation therapy, with the Stattic STAT3 inhibitor, results in reduced cells viability and proliferation, as well as sensitizing to common therapies (100). DPP, a cell-permeable porphyrin compound that prevents STAT3 dimerization, can increase the sensitivity of drug-resistant gastric cancer cells to chemotherapy (101). The combination of cisplatin with YC-1, which promotes STAT3 degradation and reduces HIF-1α protein levels, results in enhanced sensitivity of hepatocellular carcinoma cells to cisplatin and suppression of tumor growth (102). Accordingly, MDA-MB-435 metastatic breast cancer cells, expressing high levels of Y-P STAT3 and of its target Bcl-2, are highly resistant to chemotherapy-induced apoptosis. Blockade of STAT3 activation with the EGFR and JAK2 kinase inhibitors PD16839 or AG490 re-establishes sensitivity to taxol and adriamycine (103).

The development of pathway-targeted cancer drugs has raised hopes of personalized intervention via the inhibition of specific oncogenic pathways, but the dramatic responses often obtained are invariably hampered by the onset of resistance. Interestingly, drug resistance to RTK inhibitors in lung adenocarcinoma cells was recently shown to involve the activation of Y-P STAT3 via repression of MEK activity (104). These findings suggest that inhibition of this STAT3 feedback loop may enhance responses to a wide range of drugs targeting oncogene addiction.

Tumors displaying constitutively Y-P and/or S-P STAT3 may also be protected from drugs acting by generating oxidative stress (Figure 2), since both nuclear and mitochondrial STAT3 lead to decreased ROS levels and increased antioxidant factors (see above). On the other hand, ROS can stimulate the JAK2/STAT3 pathway through the induction of a positive ROS/IL-6/JAK2/STAT3 feedback during starvation-induced autophagy of cancer cells (105). Accordingly, oxidative stress triggers STAT3 activation in several cell types (106–108), and ROS are involved in EGF-induced STAT3 phosphorylation in prostate cancer (33), leading to increased pro-tumorigenic action. Finally, the oxidation of conserved STAT3 cysteines was shown to negatively modulate its activity on a subset of target genes, reducing proliferation but enhancing resistance to oxidative stress in breast cancer cells (109). STAT3 activity can also be down-modulated by cysteine glutathionylation (110). Excessive oxidative stress can also inhibit caspases activity (111, 112) and drug-induced apoptosis (113, 114). Taken together, these observations suggest that, in certain cases, excessive ROS production triggered by drug treatment may even interfere with treatment effectiveness and contribute to the development of drug resistance. Even if no direct correlation between drug resistance and energy metabolism has been so far shown, it is likely that STAT3-driven metabolic features may play crucial roles in inducing at least some of the events described above. Further studies will be required to shed light on this issue.

The recently uncovered activities of nuclear and mitochondrial STAT3 on the balance between aerobic glycolysis and oxidative phosphorylation and on ROS production suggest that agents blocking STAT3 functions at different levels may be beneficial in association with agents acting via ROS production. Indeed, inhibition of STAT3 activity was shown to increase responses of pancreatic cancer cells to gemcitabine, a ROS generating agent (115).

## Concluding Remarks

Intact apoptotic processes are required for anti-neoplastic agents to exert their optimal cytotoxic activity. Mitochondria, acting as hubs for signals that regulate energy metabolism, ROS production, and apoptotic processes, are a preferential site for multiple alterations during cancer, taking the center stage as targets for wide-spectrum cancer therapies (116, 117). In this light, the novel canonical and non-canonical STAT3 functions on energy metabolism and oxidative stress may provide targets for developing specific treatments to associate to chemical, radiation-mediated, or targeted therapies in order to overcome drug resistance and to prevent the emergence of resistant clones. However, since STAT3 deletion leads to decreased mitochondrial function and increased oxidative stress, a selective inhibition of its nuclear functions preserving mitochondrial activity may be beneficial in treating Y-P STAT3-driven cancer/drug resistance. On the other hand, mitochondrial S-P STAT3 is crucial to cell survival in RAS-transformed cells, where specific inhibition of this form may be desirable. Thus, the characterization of Y-P versus S-P STAT3 levels in tumor cells may lead to personalized intervention with respect to STAT3 activity.

## Conflict of Interest Statement

The authors declare that the research was conducted in the absence of any commercial or financial relationships that could be construed as a potential conflict of interest.
